# Sexually transmitted infections, the epidemic that persists after the COVID-19 pandemic: an analysis of the primary care electronic health records covering about 5 million people in Catalonia

**DOI:** 10.1186/s12875-024-02395-4

**Published:** 2024-05-04

**Authors:** Carolina Guiriguet, Mireia Alberny, Núria Mora, Oriol Rebagliato, Carme Roca, Francesc Fina, Mireia Fàbregas, Mència Benítez, Mariam de la Poza, Manuel Medina, Souhel Flayeh, David Pedrazas, Montserrat Sabatés, Ermengol Coma

**Affiliations:** 1 Primary Care Services Information Systems (SISAP), Institut Català de La Salut (ICS), Gran Via de Les Corts Catalanes, 587. 08007 Barcelona, Spain; 2 Gotic Primary Care Centre, Institut Català de La Salut (ICS), Barcelona, Spain; 3 Medical Management of Primary Care Services, STI/HIV Area, Institut Català de La Salut (ICS), Barcelona, Spain; 4 El Clot Primary Care Centre, Institut Català de La Salut (ICS), Barcelona, Spain; 5 University of Barcelona, Barcelona, Spain; 6 Doctor Carles Riba Primary Care Centre, Institut Català de La Salut (ICS), Barcelona, Spain

**Keywords:** Sexually transmitted diseases, Covid-19, Primary care, Epidemiology

## Abstract

**Background:**

The aim of our study is to analyse the trends in the diagnosis of sexually transmitted infections (STIs) during the COVID-19 pandemic.

**Methods:**

We conducted an observational retrospective population-based study using data from primary care electronic health records spanning from January 2016 to December 2022 (involving 5.1 million people older than 14 years). We described the daily number of new STI diagnoses from 2016 to 2022; as well as the monthly accumulation of new STI diagnoses for each year. We compared the monthly averages of new diagnoses in 2019, 2020, 2021 and 2022 using the T-test. Finally, we performed a segmented regression analysis of the daily number of STI diagnoses.

**Results:**

We analysed 200,676 new STI diagnoses. The number of diagnoses abruptly decreased coinciding with the lockdown. Overall in 2020, we observed a reduction of 15%, with higher reductions for specific STIs such as gonorrhoea (-21%), chlamydia (-24%), and HIV (-31%) compared to 2019. Following this drastic drop, which was temporarily associated with the lockdown, we observed a rapid rebound. In 2021, the number of STI diagnoses was similar to that of 2019. Notably, we found a considerable increase in 2022, particularly for non-specific STI, which lack laboratory confirmation (67% increase). HIV was the only STI with a reduction of up to -38% in diagnoses at the end of 2022 compared to 2019.

**Conclusions:**

After a significant reduction in 2020, the number of STIs recorded in primary care rapidly rebounded, and the current trend is similar to that of 2019, except for HIV. These findings underscore the dynamic impact of the COVID-19 pandemic on STI diagnoses and highlight the importance of ongoing monitoring and public health interventions in the post-pandemic period.

**Supplementary Information:**

The online version contains supplementary material available at 10.1186/s12875-024-02395-4.

## Background

The coronavirus diseases 2019 (COVID-19) pandemic has become one of the greatest public health challenges in recent times [1]. The first cases in Europe were identified in France on 24 January 2020 [2], and the first official case in Catalonia (Spain) was reported a month later on 25 February. During the first wave of the COVID-19 pandemic, many countries established a national lockdown in an attempt to control the spread of the virus, leading to a disruption of usual health care delivery systems [3, 4]. Outpatient care was restricted, and non-emergency consultations needed to be postponed. In Spain, the lockdown began on 14 March and ended on 21 June 2020 [5], encompassing a de-escalation strategy from 28 April, allowing limited social contact. Face-to-face visits were dramatically reduced during the lockdown period with variability in their subsequent recovery. Therefore preventive care, such as screening, diagnosis and testing were also affected [6–8]. These activities involve common health problems like chronic diseases, malignant neoplasms or other infections such as sexually transmitted ones [9, 10]. In October 2020, additional social restrictions were implemented in Spain, as in many European countries, including a time limit in bars and restaurants schedules, restrictions in cultural and mass events, different levels of perimeter lockdowns, and at nighttime curfew. All these strict measures may have reduced casual sexual encounters and, therefore, the incidence of sexually transmitted infections (STIs). National and international circulation and tourism have also been restricted. Tourism has been related to more sexual contacts and STIs [11].

In the last few years, the incidence of STIs has increased in Europe, with more than half a million notifications annually in the European Union and the UK. Chlamydia is the most frequently reported STI in Europe, followed by gonorrhoea, with an increasing trend [12]. In Spain and Catalonia, the incidences of these STI are among the highest in Europe, as well as for syphilis [12]. In 2015, an Integrated System of Epidemiological Surveillance of AIDS/HIV and STI in Catalonia was created. It integrates strategic sources of information, such as the system of notifiable diseases and the Microbiological Notification System of Catalonia. Syphilis, HIV, trichomoniasis, gonorrhoea, chlamydia genital infection, and *Lymphogranuloma venereum* are subject to mandatory and individualised notifications [13]. This notification system is integrated into the electronic health records (EHR) software implemented in primary health care throughout Catalonia. STIs are managed in primary care practices (PCP), where laboratory tests are available for etiological diagnosis. Sexually transmitted diseases consultants support general practitioners.

The COVID-19 pandemic has led to a reduction in new diagnoses of common diseases, including notified infectious diseases, such as other respiratory transmitted diseases and STI [14, 15]. The decline in STI infections during the early months of the pandemic has been described in previous studies, showing a variety of results. A reduction in the number of STI diagnoses during 2020 compared to 2019, especially chlamydia, has been reported in several countries [9, 16, 17, 18, 19]. However, after the lockdown, some increase or rebound in STI diagnoses has also been observed in late 2020 and early 2021 [16–20]. There are still some questions to resolve regarding the evolution of STIs during and after the pandemic, as many research studies still have limited follow-up. In this context, controversy exists in published data about the duration and magnitude of this reduction -or even increase in some cases- and its underlying causes [21].

The aim of our study is to describe the trends of registered new STI diagnoses in Catalonia, a northeastern region of Spain with 7.7 million inhabitants, by analysing data from primary care EHR, more than 3 years after the onset of the COVID-19 pandemic.

## Methods

We conducted a retrospective longitudinal study of STIs registered in primary care practices. The data were extracted from the primary care EHR of the Catalan Institute of Health (Institut Català de la Salut, ICS for its Catalan initials). The ICS is the main primary care provider in Catalonia. It manages 3 out of 4 PCPs in the Catalan public health system and covers about 5.8 million people.

We included all patients older than 14 years old with an STI diagnosis recorded in the EHR (see ICD-10 codes in Supplementary Table 1). This encompasses specific STIs as well as non-specific infections, where diagnoses lack laboratory confirmation.

The study period spanned from January 2016 to December 2022. We divided this period into three for the analysis: pre-COVID period from January 2016 to March 13, 2020; lockdown from March 14, 2020, to June 20, 2020, which encompasses the lockdown in Spain and various stages of the de-escalation; and COVID-19 period from June 21, 2020, to December 31, 2022, covering the entire pandemic period following the lockdown in Spain. Throughout this last period, several non-pharmaceutical interventions (NPIs) were implemented, with some changes in certain months that included the closure of non-essential activities (October 2020), at night curfew (during specific periods of 2021), mobility restrictions, test and trace measures, mask mandates, etc. In the second half of 2022,mask mandates only remained in place in certain settings.

Analyses were conducted globally and stratified by sex, age groups (15–29 years old, 30–59 years old, and older than 59 years old), type of infection, socio-economic status, and rurality. We assessed the socioeconomic status using the validated MEDEA deprivation index [22]. We categorised the MEDEA deprivation index into four groups, with the 1st and 4th groups representing the least and most deprived areas, respectively. Rural areas were categorised separately and defined as areas with less than 10,000 inhabitants and a population density lower than 150 inhabitants/km^2^.

### Statistical analysis

We depicted the daily count of new STI diagnoses from 2016 to 2022 and the monthly cumulative count of new STI diagnoses for each year. We performed a comparison of monthly averages for new diagnoses between the years 2019, 2020, 2021, and 2022 using the T-Student test.

### Poisson segmented regression analysis

To assess the impact of the COVID-19 pandemic on the evolution of daily new STI diagnoses, a Poisson segmented regression analysis was performed with the following formulation:

$$log \left(Y\right) \sim beta\_0+{beta\_1 }^{*} time+{beta\_2}^{ *} pandemic+{beta\_3}^{ *} time\_covid+{beta\_4}^{ * }time\_post\_lockdown+error$$

where.

*Y* represents the 7-day moving average of daily new STI diagnoses,

*Time* denotes the day of diagnosis,

*Pandemic* is a dummy variable with a value of 1 if the time is later than March 14, 2020, and 0 otherwise,

*Time_covid* is the number of days since March 14, 2020, and.

*Time_post_lockdown* is the number of days since June 21, 2020.

The slope associated with the pre-pandemic period is estimated by *beta_1*, while the slope associated with the lockdown period is estimated by *beta_1* + *beta_3* and the slope associated with the post-confinement period is estimated by *beta_1* + *beta_3* + *beta_4*. *Beta_2* assesses the effect of the pandemic on the daily count of new diagnoses.

The statistical significance level of all tests is set at alpha = 0.05.

All analyses were performed using R, version 3.5.1 [23].

## Results

### Results overview

Between January 2016 and December 2022, a total of 200,676 new STI diagnoses were recorded in the primary care EHR. The male proportion ranged from 56 to 57% for all years, except for 2020, where it slightly increased to 59%. The socioeconomic distribution remained similar throughout the study period. The percentage of non-specific STI and non-specific urethritis increased (from 6.66% and 19.98% in 2019 to 9.69% and 22.46% in 2022, respectively), while syphilis and HIV experienced slight declines (see Table 1).

**Table 1:** Epidemiological characteristics of the STI diagnoses in Catalonia by year since January 2016 to December 2022

	**2016**		**2017**		**2018**		**2019**		**2020**		**2021**		**2022**	
**Total number of STI diagnoses**	22,721		24,813		27,146		31,334		26,691		32,096		35,875	
**Monthly mean number of STI**	1893.4		2067.75		2262.2		2611.2		2224.3		2674.7		2989.6	
	**N**	**%**	**N**	**%**	**N**	**%**	**N**	**%**	**N**	**%**	**N**	**%**	**N**	**%**
**Age groups**														
15–29 years	8983	39.54%	10,259	41.35%	11,892	43.81%	14,461	46.15%	12,279	46.00%	14,720	45.86%	16,642	46.39%
30–59 years	12,303	54.15%	13,091	52.76%	13,875	51.11%	15,316	48.88%	13,069	48.96%	15,769	49.13%	17,324	48.29%
> 59 years	1435	6.32%	1463	5.90%	1379	5.08%	1557	4.97%	1343	5.03%	1607	5.01%	1909	5.32%
**Sex**														
Women	9692	42.66%	10,745	43.30%	11,722	43.18%	13,721	43.79%	10,921	40.92%	13,634	42.48%	15,354	42.80%
Men	13,029	57.34%	14,068	56.70%	15,424	56.82%	17,613	56.21%	15,770	59.08%	18,462	57.52%	20,521	57.20%
**Socioeconomic status**														
Rural areas	3601	15.85%	3811	15.36%	4403	16.22%	4997	15.95%	4355	16.32%	5380	16.76%	6023	16.79%
Urban least deprived	5796	25.51%	6555	26.42%	7000	25.79%	8292	26.46%	6843	25.64%	8256	25.72%	9626	26.83%
Urban 2Q	3360	14.79%	3582	14.44%	3902	14.37%	4430	14.14%	3831	14.35%	4520	14.08%	5042	14.05%
Urban 3Q	4595	20.22%	4963	20.00%	5430	20.00%	6040	19.28%	4996	18.72%	6098	19.00%	6808	18.98%
Urban most deprived	5369	23.63%	5902	23.79%	6411	23.62%	7575	24.18%	6666	24.97%	7842	24.43%	8376	23.35%
**STI**														
Syphilis	909	4.00%	966	3.89%	1078	3.97%	1239	3.95%	1042	3.90%	996	3.10%	1176	3.28%
Gonorrhea	936	4.12%	1264	5.09%	1555	5.73%	2167	6.92%	1709	6.40%	1988	6.19%	3125	8.71%
Chlamydia	1932	8.50%	2721	10.97%	3922	14.45%	5650	18.03%	4290	16.07%	6010	18.73%	6436	17.94%
HIV	1336	5.88%	1280	5.16%	1141	4.20%	1201	3.83%	830	3.11%	805	2.51%	742	2.07%
Genital herpes	3090	13.60%	3193	12.87%	3520	12.97%	3895	12.43%	3425	12.83%	4024	12.54%	4719	13.15%
Non-specific urethritis^a^	5773	25.41%	6418	25.87%	6057	22.31%	6260	19.98%	6430	24.09%	7113	22.16%	8059	22.46%
Non-specific STI^a^	962	4.23%	1065	4.29%	1436	5.29%	2086	6.66%	2068	7.75%	2991	9.32%	3477	9.69%
Others (anogenital warts, lymphogranuloma venereum)	7783	34.25%	7906	31.86%	8437	31.08%	8836	28.20%	6897	25.84%	8169	25.45%	8141	22.69%

All ICD-10 codes are listed in Supplementary material

^a^STIs that lack laboratory confirmation

The monthly average number of STI diagnoses increased from 1893.4 in 2016 to 2611.2 in 2019. However, in 2020, there was a 15% reduction (*p*-value < 0.05), decreasing to 2224.3. Subsequently, it rebounded to 2674.7 in 2021 and 2989.6 in 2022. Notably, the monthly average number of STI diagnoses in 2021 was comparable to that of 2019 (2.4% increase [*p*-value = 0.49], while in 2022, we observed a significant increase of 14.5% [*p*-value < 0.05]) (Supplementary Table 2).

### Trends according to type of STI

**Fig. 1:**
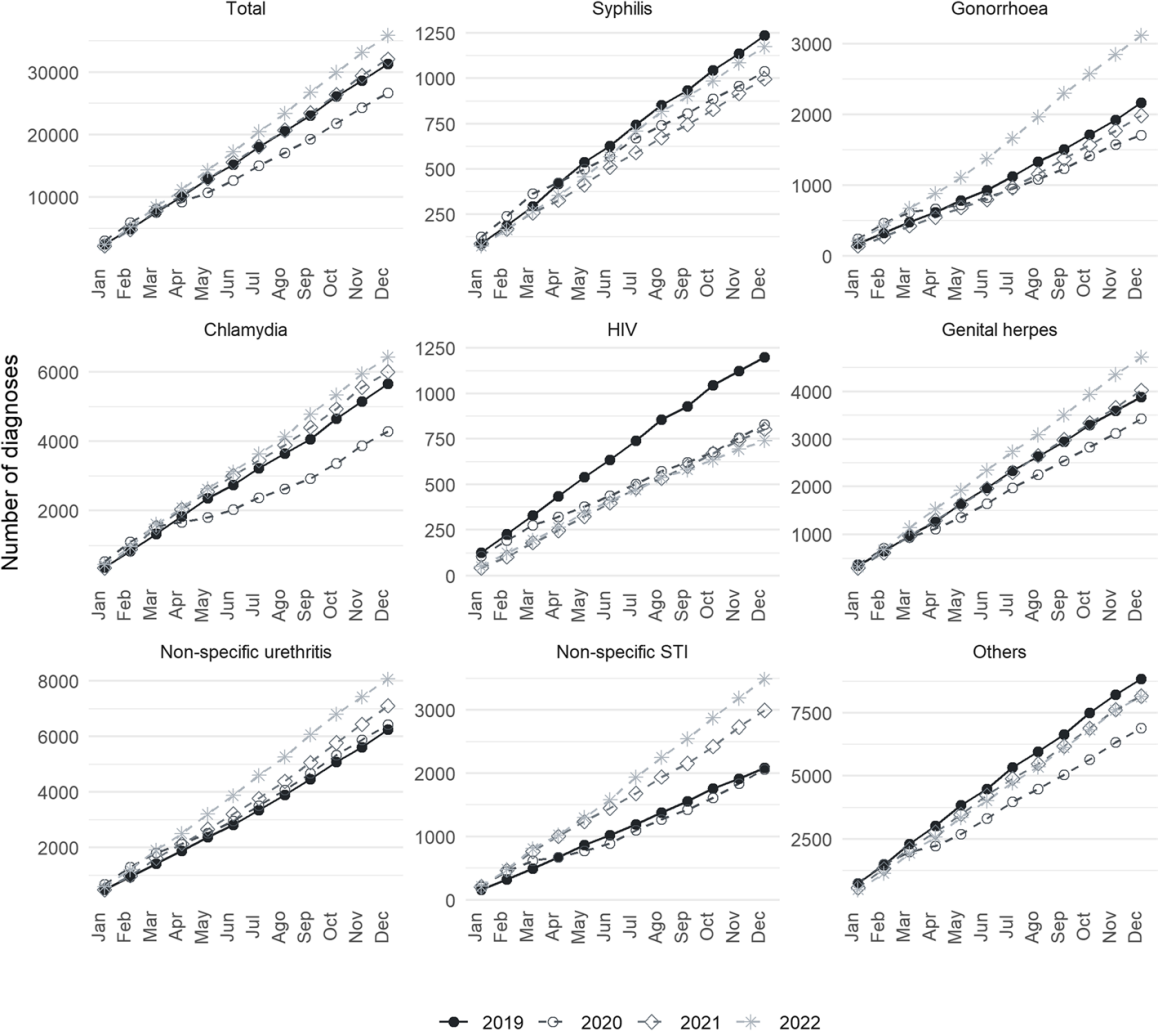
Cumulative number of STI registered diagnoses by year in Catalonia (2019–2022)

Figure 1 shows the cumulative number of STI by year since 2019. All STIs decreased in 2020 compared to 2019, a decline that began in March–April 2020 coinciding with the lockdown in Spain. Afterward, the number of diagnoses in 2020 did not reach those in 2019. However, in 2021, the number of diagnoses was similar to that of 2019, except for HIV (33% of reduction, *p*-value < 0.05), syphilis (19.6% reduction, *p*-value < 0.05) and for non-specific STIs, which significantly increased (43.4% increase in 2021 compared to 2019, *p*-value < 0.05). In2022, the same pattern was observed for HIV, which still had a significant reduction of -38.2%; while non-specific STIs and non-specific urethritis have statistically significant increases. Finally, the number of recorded diagnoses of gonorrhoea, chlamydia and genital warts increased significantly in 2022 (44%,14% and 21% respectively, *p*-values < 0.05).

### Sex and socioeconomic differences

These reductions during 2020 were observed in all age groups and both sexes, although they were greater in women (-20.41% vs -10.46%) and statistically non-significant in men and in the population between 15 and 29 years (Supplementary Table 2). In 2021, the number of diagnoses for each category slightly increased but was not statistically significantly different compared to 2019. In contrast, in 2022, we observed statistically significant increases in all age groups and sexes (Supplementary Table 2). Regarding socioeconomic status, the reduction of STI recorded diagnoses in 2020 was less pronounced in the most deprived areas, with a -17.47% reduction in the least deprived areas compared to 2019 (*p*-value: < 0.05), versus a statistically non-significant reduction of -12% in most deprived areas (*p*-value: 0.15). Supplementary Figs. 1, 2, 3, and 4 visually depict these variations.

### Segmented regression analysis

**Fig. 2:**
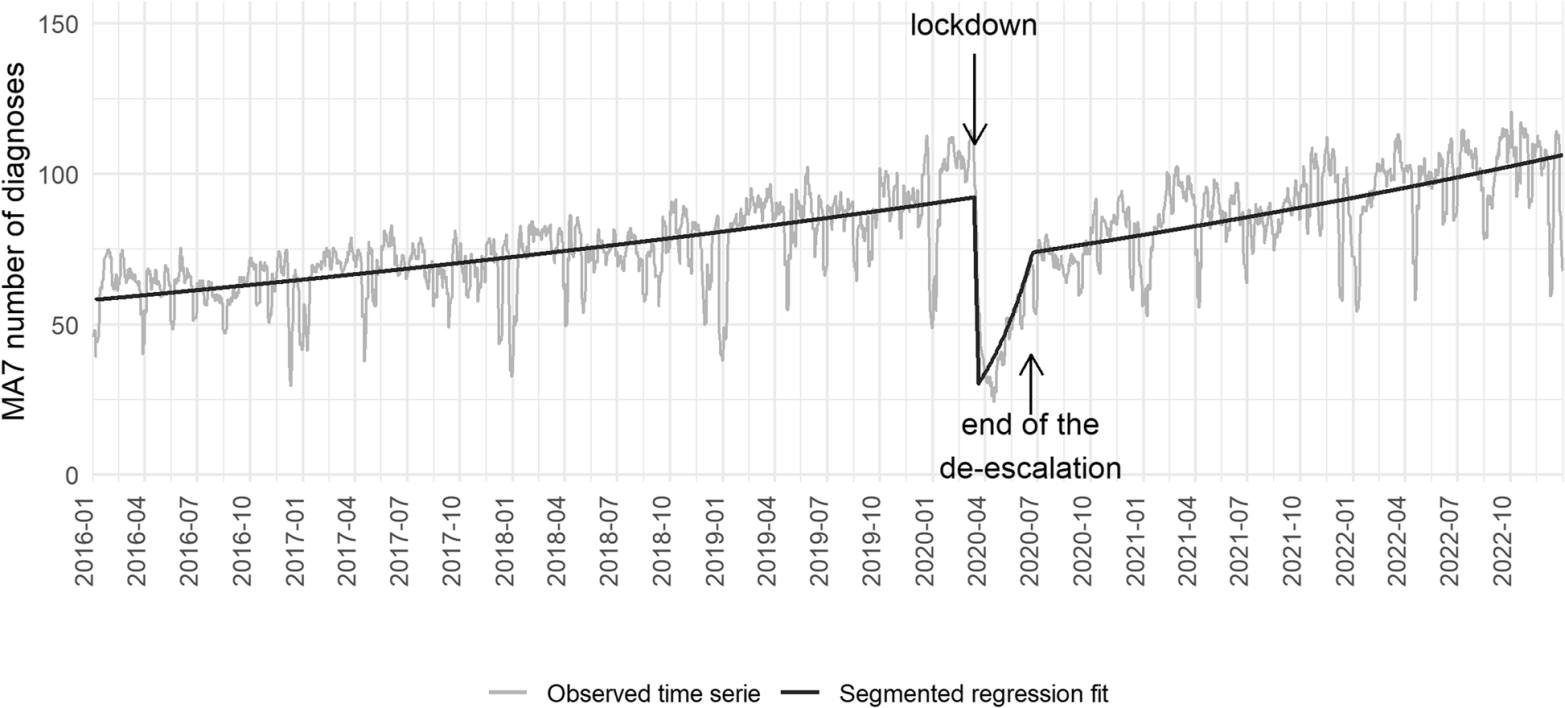
7-day moving average number of daily STI registered diagnoses since 2016 in Catalonia

Finally, Fig. 2 showcases the results of the Poisson segmented regression analysis. The increasing trend of daily STI diagnoses was abruptly interrupted on March14, 2020 (the day of the beginning of the lockdown in Spain). Subsequently, there was an increase during the de-escalation months. Post-June 2020, the trend resembled that of the pre-pandemic period, albeit with a lower number of diagnoses compared to the previous trend. Table 2 presents the coefficients of the model for the total number of STI diagnoses.

**Table 2:** Coefficients of the regression model

coefficient	Beta	Lower 95% CI	Upper 95% CI
(Intercept)	4.064057	4.05167219	4.07641025
time	0.00030032	0.00028711	0.00031353
Pandemic	-1.1444345	-1.2027806	-1.0868113
time_covid	0.00911586	0.00850071	0.00973802
time_post_lockdown	-0.0090231	-0.0096536	-0.0083995

## Discussion

Our study analyses the trends of STI diagnoses recorded in primary care EHR for over two years following the onset of the COVID-19 pandemic. We observed a significant decline in the number of STIs just before the implementation of the lockdown in Spain. The trend prior to the lockdown was on the rise in our reference area (Catalonia), aligning with the trends in the rest of Europe. In this context, the lockdown brought about a shift in the trend, as also noted in a previous study [24], more notable in women, individuals under 60 years of age, residents in less deprived urban areas, and specific types of STIs (chlamydia, gonorrhoea and HIV). Following this sharp decline, which was temporally associated with the lockdown, there was a rapid rebound, resulting in an overall reduction of 15% in 2020 compared to 2019. In 2021, the number of STI diagnoses was comparable to that in 2019 and, ultimately, we observed a significant increase in 2022. However, the substantial reduction (up to -38%) in HIV diagnoses observed in 2022 remains concerning, marking it as the sole STI with fewer diagnoses at the close of 2022 compared to 2019.

Several studies have highlighted a decline in new STI diagnoses during the lockdown period, potentially linked to the impact of social distancing measures [7, 9, 10, 14–21, 24–27]. While stringent social distancing measures might have influenced the population’s sexual behaviour, thereby affecting STI incidence [28–30], the actual cause of this reduction remains a subject of debate. It raises questions about whether the decline resulted from reduced social interaction or limited accessibility to testing.

During the six weeks of strict confinement, medical services remained operational only for urgent consultations, which could have led to delayed or missed diagnosis of less symptomatic STIs, as well as a reduction in routine screening tests and serology. Several countries in the WHO European Region reported substantial disruptions in testing supply, with volumes decreasing by over 50%, attributed to factors such as testing centre closures, reduced attendance, and laboratory overload. [7]. Moreover, a study conducted in Hungary observed a decline in chlamydia and syphilis diagnoses, while gonorrhoea diagnosis increased. Since syphilis and chlamydia are typically identified through STI screening due to their asymptomatic nature, unlike gonorrhoea, the results of this study suggest that the reduction in STI cases could be a pseudo-reduction due to an increase in undetected cases [21].

HIV and chlamydia infections showed the greatest reduction in our study. This finding surpassed the reported percentages of less than 20% in certain studies for chlamydia [9, 15–17, 27]. However, a study conducted in Arizona by Bell et al. also found a roughly 22% decline in screening chlamydia diagnosis and a 30% reduction in testing [18]. Similar reductions were described by Crane et al. in Baltimore [9], and Braunstein et al. reported an even more substantial decrease during the statewide executive order in New York compared to our study [31]. In Spain, primary care laboratories offer a multitest that includes screening for gonococcal, chlamydial, Mycoplasma genitalium, and trichomonas infections. This could account for the concordant reduction in chlamydial and gonococcal infections, with a more pronounced decrease in the former due to the higher number of asymptomatic cases detected by screening measures that were interrupted during the lockdown period and its aftermath. However, some European countries reported no significant changes in the incidence [15] or prevalence [6] of chlamydial and gonococcal infections, possibly due to less restrictive travel and social measures [32]. Gonococcal genital infection exhibited discrepancies in previous studies, showing a broad range from increased incidence [9, 14, 27, 33] (up to 56% in Taiwan [14]) to reduced new diagnoses [9, 15, 25] (up to 36.9% reduction in Greece [16]).

Furthermore, cases such as the one in Melbourne offer valuable insights. Despite maintaining routing care at sex centres, a 40% reduction in attendances, a 65% reduction in screening and a 40% reduction in non-gonococcal urethritis was observed during the closure period (March 23, 2020, to May 20, 2020), compared to earlier in the year. However, no significant differences were observed for syphilis or pelvic inflammatory disease. In our study, syphilis was among the STIs with the smallest reduction. This finding is consistent with data reported elsewhere, showing minor increases and reductions of less than 10% in syphilis trends [9, 14, 25, 27]. This may suggest that individuals with more specific clinical presentations sought medical care, leading to a specific STI diagnosis [10]. Another reason could be that the extragenital or transmissible skin lesions of syphilis may escape the use of barrier methods to prevent STIs, in contrast to the urethral discharge characteristic of gonorrhoea, which exhibited greater reductions than syphilis in the context of the COVID-19 pandemic [17, 24]. Additionally, non-specific STIs and urethritis experienced a significant increase, possibly related to the underdiagnosis of specific STIs due to restrictions affecting both laboratory and PCP accessibility.

Finally, in 2022, all STIs have resumed the upward trend observed before the COVID-19 pandemic, except for HIV, which remains the only STI with continued reductions compared to 2019. This underdiagnosis of HIV aligns with published data [14, 15, 27]. Despite the decline in diagnoses may also be attributed both to undetectable cases and to the continuity of pre-exposure prophylaxis care during the COVID-19 pandemic in Catalonia, it warrants special attention due to the associated burden of disease.

### Limitations and strengths

We acknowledge several limitations in our study. First, we conducted an ecological study, which does not allow for establishing a causal correlation between the reduction in incidence and the COVID-19 pandemic. Second, since our analysis is based on the number of diagnoses, significant changes in population structure could potentially limit the results. However, it's noteworthy that the age and gender distribution of the population has remained relatively stable throughout the study period, as indicated in previous publications by our research team [34]. Third, the use of primary care EHR could imply a potential lack of hospital diagnoses and a potential diagnoses inaccuracy, as it relies on routinely collected data. Nevertheless, the Catalan primary care EHR data have been used in several studies, contributing valuable insights under real-world conditions, including other analyses focusing on diagnoses during the pandemic [34, 35]. Additionally, in the Catalan primary care EHR, sex is categorised as a binary variable, lacking consideration for a gender perspective or sexual orientation. Finally, the lack of information on ethnicity, sexual behaviours and the use of HIV pre-exposure prophylaxis in the Catalan primary care EHR could limit the interpretation of our findings.

The study also has notable strengths. Our analysis surpasses the short-term effects of the initial months of COVID-19, extending for more than two years—a duration that distinguishes it from studies concentrating solely on the lockdown period or a few months afterward. We also assessed socioeconomic differences, particularly relevant for STIs and vulnerable populations. Furthermore, while many studies focused on specific mandatory notifiable STIs, our research offers a comprehensive overview of the most common STIs managed in primary health care. Additionally, we included data on non-specific STIs, a relevant addition considering the potential under-registration of new specific STI diagnoses due to limited access to laboratory tests. Finally, given that ICS manages about 75% of primary care practices in Catalonia, our results are generalizable, and our methodology could be applied in other settings employing EHR.

## Conclusions

After a significant decline in the number of STIs recorded in the primary care EHR associated with the 2020 lockdown and related measures, there was a rapid rebound and the current trend is similar to that of 2019. This reduction could be attributed to decreased testing, as non-specific STIs were not significantly reduced. Additionally, in 2022, we observed an increase in the numbers of certain STIs (chlamydia, gonorrhoea, and genital herpes), which should be taken into account by healthcare authorities. Moreover, there remains a nearly 40% reduction in HIV diagnoses compared to 2019. Urgent policy interventions are necessary to address consequences of this potential underdiagnosis. Furthermore, long-term studies are needed to evaluate future impacts. A lesson learned from the COVID-19 pandemic underscores the importance of maintaining access to medical care for STI-related consultations and promoting self-testing and home-based screening strategies [36] for vulnerable individuals and those engaging in high-risk sexual behaviour.

### Supplementary Information

**Supplementary Material 1.**

**Supplementary Material 2.**

**Supplementary Material 3.**

## Data Availability

The datasets used during the current study are available upon reasonable request to the corresponding author.

## Abbreviations

95%CI: 95% Confidence interval
COVID-19: Coronavirus disease 2019
EHR: Electronic health records
ICS: Catalan Institute of Health (for its Catalan initials)
ICD-10: International Classification of Diseases 10th revision
STI: Sexually transmitted infections
