# Supplementary material for: Sexually transmitted infections, the epidemic that persists after the COVID-19 pandemic: an analysis of the primary care electronic health records covering about 5 million people in Catalonia
Source: BMC Prim Care. 2024 May 4;25:150. doi: 10.1186/s12875-024-02395-4 (PMC11069189; doi:10.1186/s12875-024-02395-4)
Supplement: Supplementary file 1 — Supplementary Material 1. [file 12875_2024_2395_MOESM1_ESM.docx]

**Supplementary Figure 1.** Difference in the cumulative number of STI registered diagnoses compared to 2019

**
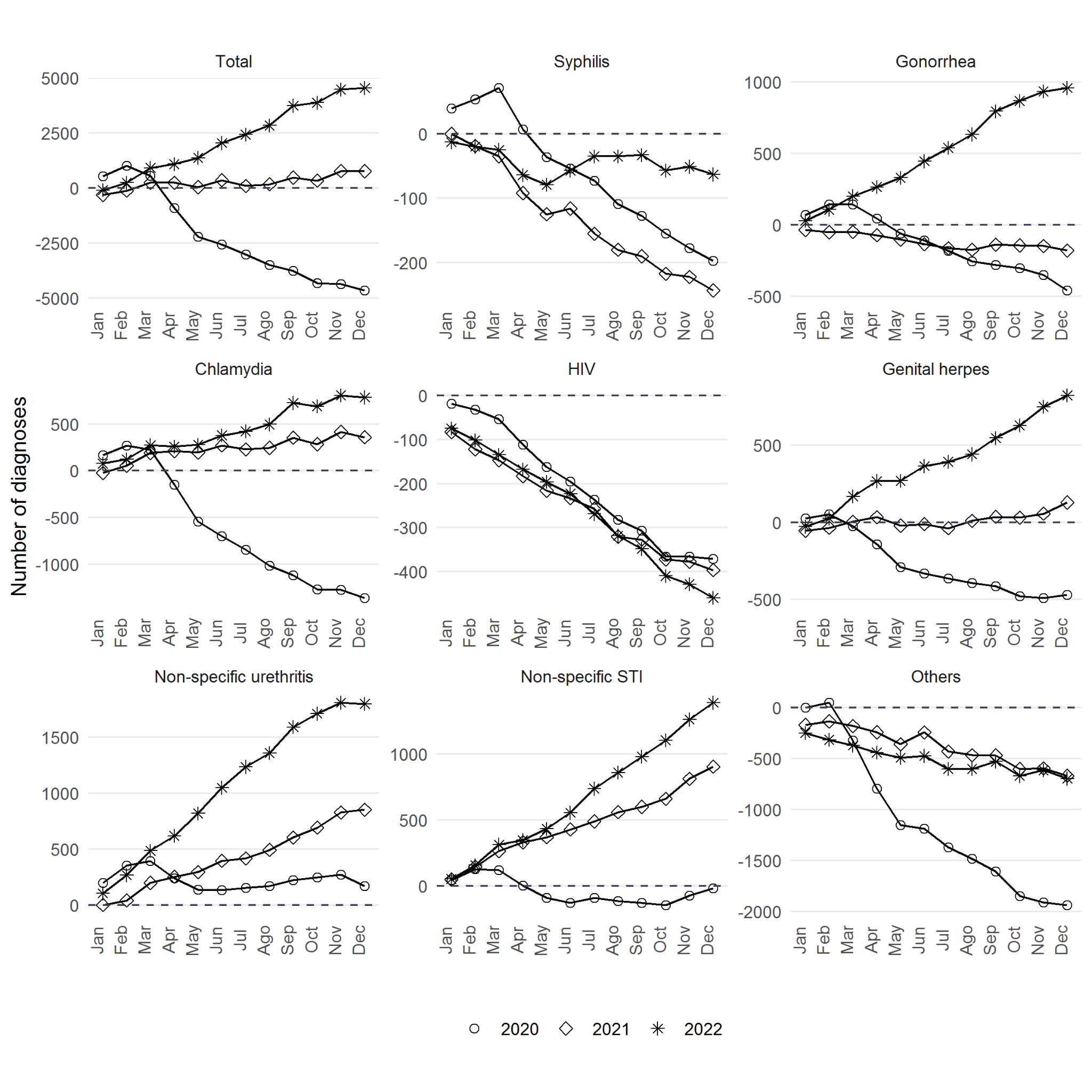
**

**Supplementary Figure 2** Difference in the cumulative number of STI registered diagnoses compared to 2019 by age group

**
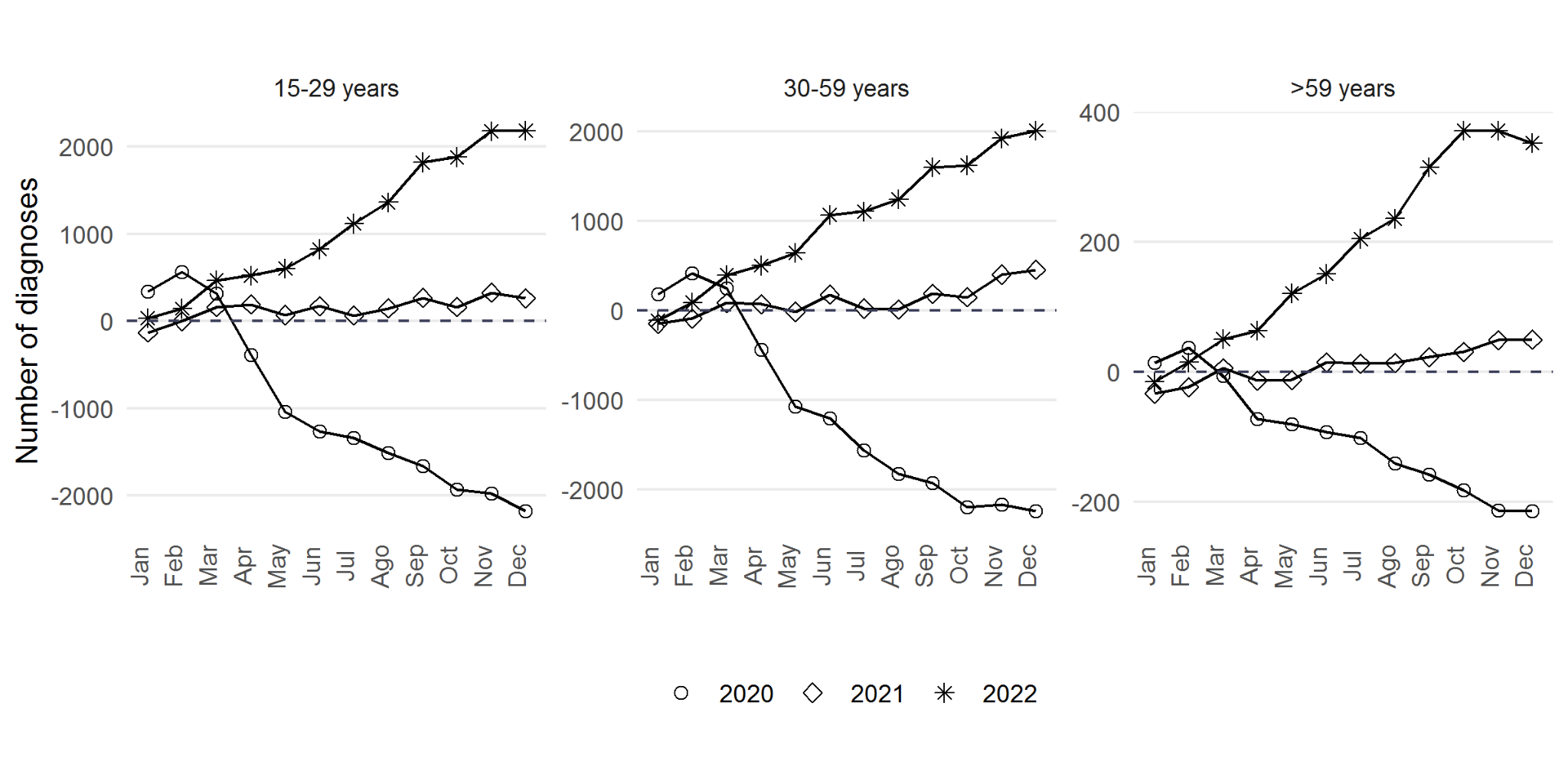
**

**Supplementary Figure 3** Difference in the cumulative number of STI registered diagnoses compared to 2019 by sex

**
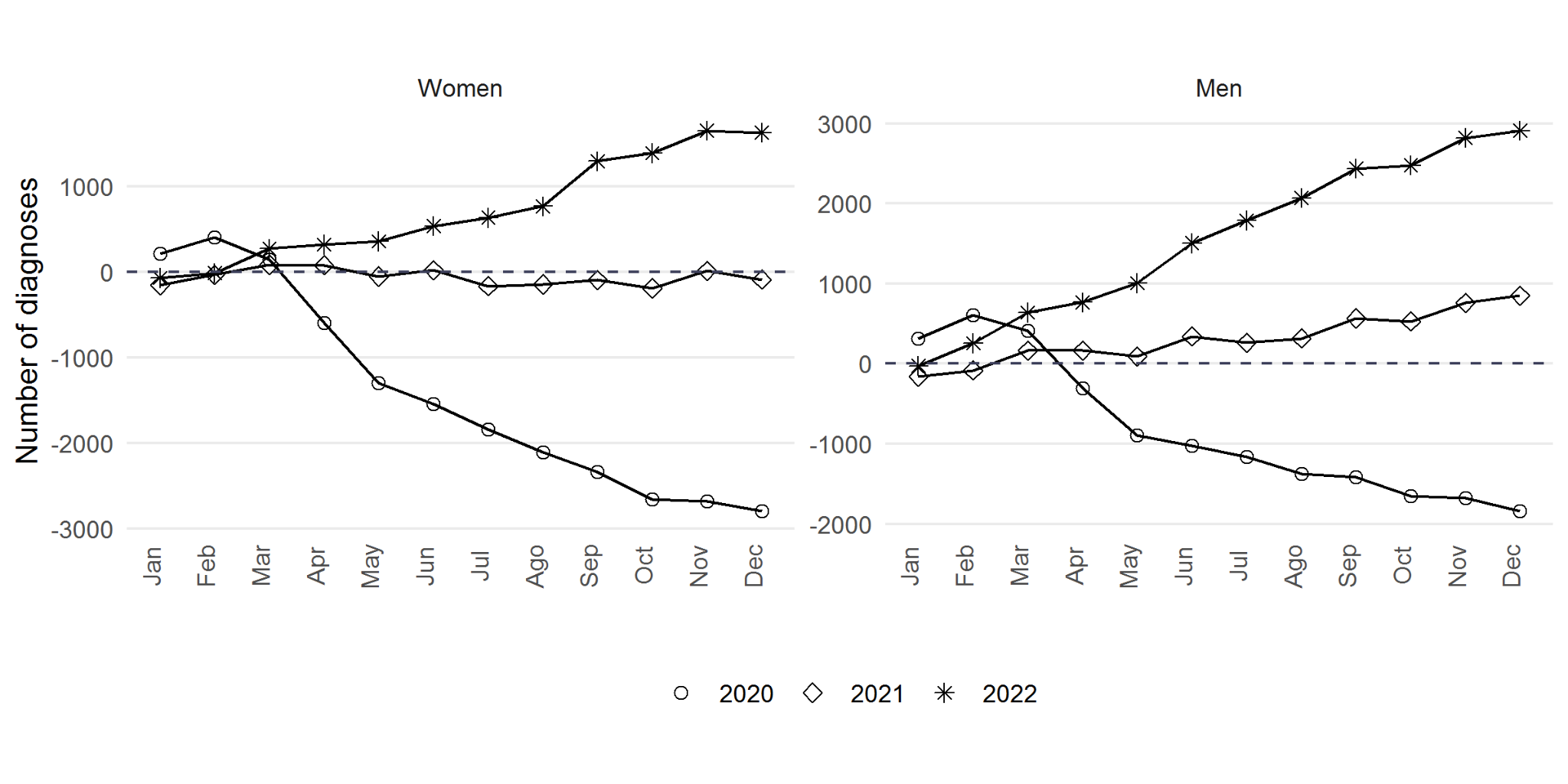
**

**Supplementary Figure 4** Difference in the cumulative number of STI registered diagnoses compared to 2019 by socioeconomic status

**
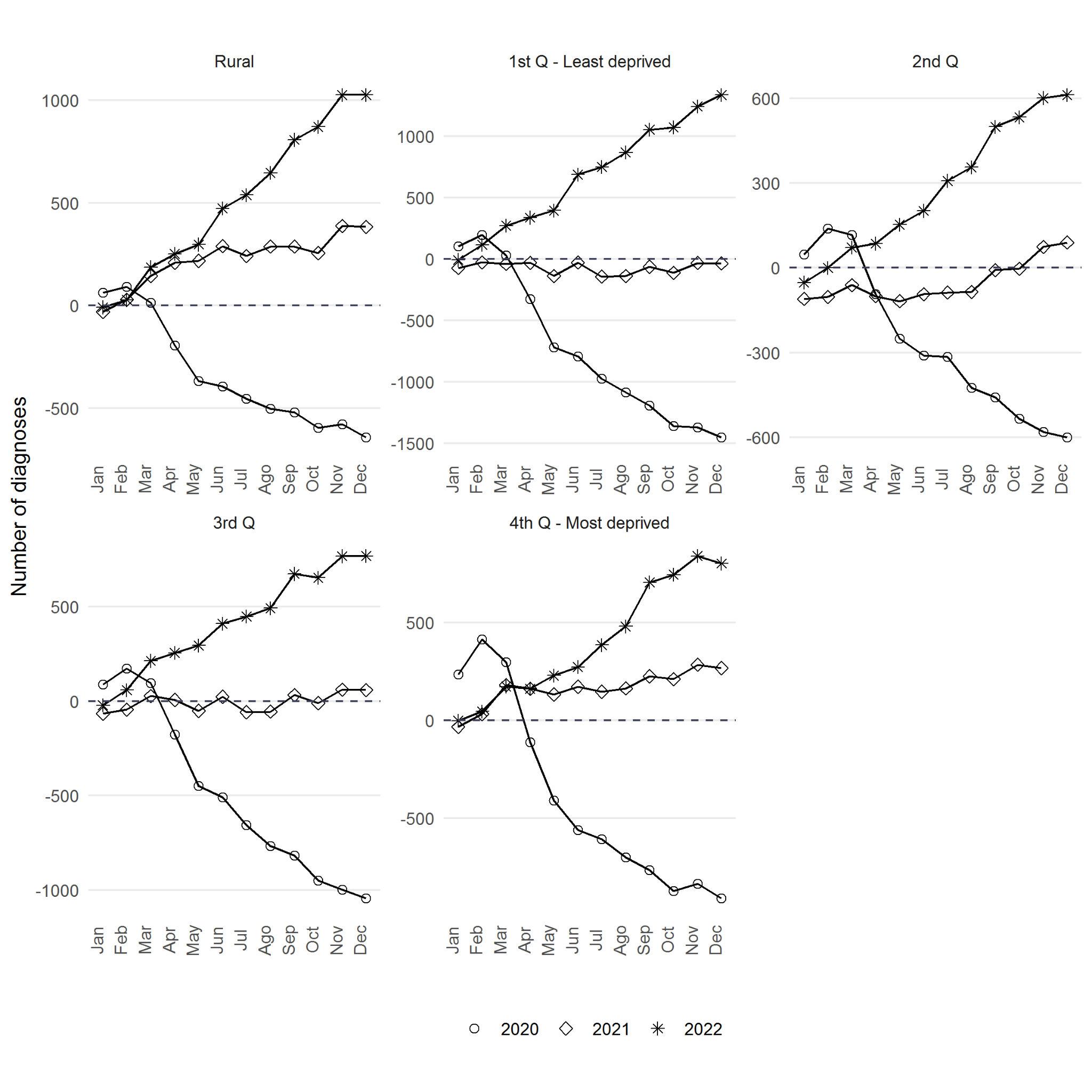
**
