# Supplementary material for: Sexually transmitted infections, the epidemic that persists after the COVID-19 pandemic: an analysis of the primary care electronic health records covering about 5 million people in Catalonia
Source: BMC Prim Care. 2024 May 4;25:150. doi: 10.1186/s12875-024-02395-4 (PMC11069189; doi:10.1186/s12875-024-02395-4)
Supplement: Supplementary file 2 — Supplementary Material 2. [file 12875_2024_2395_MOESM2_ESM.docx]

**Supplementary Table 1. Sexually transmitted infections included in the study and corresponding ICD-10 codes**

| **Description** | **ICD-10 code** |
| --- | --- |
| Anogenital warts | A63.0 |
| Syphilis | A51 |
|  | A51.0 |
|  | A51.1 |
|  | A51.2 |
|  | A51.3 |
|  | A51.4 |
|  | A51.5 |
|  | A51.9 |
|  | A53 |
|  | A53.0 |
|  | A53.9 |
| Gonococcal infection | 098.2 |
|  | A54 |
|  | A54.0 |
|  | A54.1 |
|  | A54.2 |
|  | A54.3 |
|  | A54.4 |
|  | A54.5 |
|  | A54.6 |
|  | A54.8 |
|  | A54.9 |
|  | M71.10 |
|  | K67.1 |
|  | M73.0 |
|  | N74.3 |
| Sexually transmitted chlamydial diseases | A56 |
|  | A56.0 |
|  | A56.1 |
|  | A56.2 |
|  | A56.3 |
|  | A56.4 |
|  | A56.8 |
|  | N74.4 |
| Human immunodeficiency virus infection/disease | Z21 |
|  | B20 |
|  | B20.0 |
|  | B20.1 |
|  | B20.2 |
|  | B20.3 |
|  | B20.4 |
|  | B20.5 |
|  | B20.6 |
|  | B20.7 |
|  | B20.8 |
|  | B20.9 |
|  | B21 |
|  | B21.0 |
|  | B21.1 |
|  | B21.2 |
|  | B21.3 |
|  | B21.7 |
|  | B21.8 |
|  | B21.9 |
|  | B22 |
|  | B22.0 |
|  | B22.1 |
|  | B22.2 |
|  | B22.7 |
|  | B23 |
|  | B23.0 |
|  | B23.1 |
|  | B23.2 |
|  | B23.8 |
|  | B24 |
|  | F02.4 |
| Trichomoniasis | A59 |
|  | A59.0 |
|  | A59.8 |
|  | A59.9 |
| Anogenital herpesviral infections | A60 |
|  | A60.0 |
|  | A60.1 |
|  | A60.9 |
| Chlamydial lymphogranuloma (venereum) | A55 |
| Unspecified sexually transmitted diseases | A64 |
|  | A63 |
|  | A63.8 |
| Unspecified urethritis | N34.0 |
|  | N34.1 |
|  | N34.2 |
|  | N34.3 |
